# Serum exocrine pancreas enzymes are biomarkers of immunotherapy response in new-onset type 1 diabetes

**DOI:** 10.3389/fendo.2024.1497373

**Published:** 2024-11-29

**Authors:** Brittany Sorensen Bruggeman, Savanna Gornisiewicz, Rhonda Bacher, Kieran McGrail, Martha Campbell-Thompson, Clive Wasserfall, Laura M. Jacobsen, Mark Atkinson, Michael J. Haller, Desmond A. Schatz

**Affiliations:** ^1^ Division of Endocrinology, Department of Pediatrics, University of Florida, Gainesville, FL, United States; ^2^ College of Medicine, University of Florida, Gainesville, FL, United States; ^3^ Department of Biostatistics, University of Florida, Gainesville, FL, United States; ^4^ Department of Pathology, Immunology, and Laboratory Medicine, University of Florida, Gainesville, FL, United States

**Keywords:** type 1 diabetes, exocrine pancreas, immunotherapy, thymoglobulin, granulocyte colony stimulating factor, pancreatic alpha-amylase, lipase, trypsin

## Abstract

**Introduction:**

The immune-mediated destruction of insulin-producing β-cells characterizes type 1 diabetes. Nevertheless, exocrine pancreatic enzymes, including amylase, lipase, and trypsin, are also significantly reduced in type 1 diabetes. With an immunotherapy now approved to treat early-stage type 1 diabetes, biomarkers to delineate response to treatment are needed. No study has yet evaluated whether serum exocrine pancreatic enzymes could delineate immunotherapy responders and non-responders.

**Methods:**

In this novel study, we sought to identify longitudinal trends in the most commonly measured circulating exocrine enzymes before and after treatment with anti-thymocyte globulin (ATG) and pegylated granulocyte colony-stimulating factor (GCSF) in individuals with new-onset type 1 diabetes (n=34). We defined response to immunotherapy as participants with at least 60% of baseline area under the curve (AUC) C-peptide levels after a 2-hour mixed meal tolerance test (MMTT) at two years post-treatment. In the overall study (n=89), 42% of treated and 17% of placebo participants met this definition. Due to constraints of sample availability, we compared longitudinal serum amylase, lipase, and trypsin levels in a subset of responders to therapy (n=4-6), placebo “responders” (n=2), treated non-responders (n=16), and placebo non-responders (n=10).

**Results:**

There were no differences in amylase levels between groups at baseline or six months post-treatment. Baseline levels of lipase and trypsin tended to be lower in responders; however, these variations were not significant in this small study sample. Lipase and trypsin improved to 115% of baseline in responders to immunotherapy six months after treatment and declined to 80-90% of baseline in non-responders and placebo participants (p=0.03). This difference was not present before the six-month time point.

**Discussion:**

Our findings provide preliminary evidence that the exocrine pancreatic enzymes lipase and trypsin may be useful biomarkers of response to immunotherapy in type 1 diabetes. Further studies with larger numbers of participants are warranted.

## 1 Introduction

Type 1 diabetes (T1D) is characterized by autoimmune destruction of insulin-producing β-cells within pancreatic islets. However, the underlying pathogenesis of T1D is not yet fully understood. In healthy individuals, the exocrine pancreas, which comprises upwards of >85% of pancreatic mass, increases in size, and serum exocrine enzymes lipase and trypsin trend upwards throughout childhood and early adulthood (1–3). These enzymes are produced by pancreatic acinar cells; serum levels are reduced in diseases that cause exocrine atrophy including cystic fibrosis and chronic pancreatitis and increased in states of acute exocrine tissue damage including acute pancreatitis (4–6). Serum amylase, while frequently used for the diagnosis of acute pancreatitis, is not as specific an indicator of pancreatic disease and does not have the same gradual increase over time (2–4). There has been substantial interest in the role of the exocrine pancreas in T1D development after a study within the Network for Pancreatic Organ Donors with Diabetes (nPOD) showed that pancreas weight was reduced in non-diabetic organ donors with positive islet autoantibodies (at risk for clinical T1D) and was confirmed in subsequent research (7–9). In individuals with pre-clinical and clinical stages of T1D, pancreas size and serum and stool exocrine enzymes are significantly decreased (7–13). Most recently, Mendelian randomization studies have demonstrated that increased circulating pancreatic enzymes play a protective role in T1D susceptibility (14, 15).

Despite this progress in understanding the exocrine pancreas in T1D, no study has yet evaluated the effect of immune therapy on circulating exocrine enzymes. In this era of clinically available T1D-directed immunotherapy, biomarkers delineating response to therapy, both prospectively and in the year post-treatment, are urgently needed to further our goal of treating the right patient with the right therapy at the right time in T1D progression (16). In an era of biomarker-adaptive clinical trial designs within other fields, the benefit of early identification of response to therapy is clear, with treatment guided by indicators of therapeutic response (17). Additionally, changes in serum exocrine enzymes after treatment with immunotherapy may inform hypotheses regarding the etiology of exocrine pancreatic dysfunction in the early T1D period (13, 18). If exocrine dysfunction results from autoimmune or inflammatory destruction or insulinopenia, treatment with an immunomodulatory agent that halts the autoimmune attack or prolongs native insulin production could prevent or reverse the impact on the exocrine pancreas (13).

In this study, we sought to identify longitudinal trends in circulating amylase, lipase, and trypsin before and after treatment with anti-thymocyte globulin (ATG) and pegylated granulocyte colony-stimulating factor (GCSF) in patients with new-onset T1D. We hypothesized that effective immunotherapies in new-onset T1D would improve exocrine and endocrine pancreatic function measures.

## 2 Materials and methods

### 2.1 Study population

Study participants provided written informed consent (and assent in the case of minors) before being enrolled in the trial, as approved by independent ethics committees or Institutional Review Boards (IRBs) (19). A subset of previously collected serum obtained in conjunction with a concluded multisite, three-arm, randomized, placebo-controlled, double-blind Type 1 Diabetes TrialNet trial (TN19): “Antithymocyte Globulin (ATG) and pegylated granulocyte colony-stimulating factor (GCSF) in New Onset Type 1 Diabetes” (ClinicalTrials.gov NCT02215200) was analyzed for exocrine pancreatic markers amylase, lipase and trypsin. Participants aged 12-45 years with new-onset T1D were randomized to three treatment groups: low-dose ATG (2.5 mg/kg for one dose), low-dose ATG + pegylated GCSF (2.5 mg/kg for one dose + 6 mg subcutaneously every 2 weeks for 6 doses), and placebo, as previously described (19).

Responders to immunotherapy were defined as participants with at least 60% of baseline area under the curve (AUC) C-peptide levels after a 2-hour mixed meal tolerance test (MMTT) at two years post-treatment (20). In the overall study (n=89), 42% of treated and 17% of placebo participants met this definition. Due to constraints of sample availability, we compared longitudinal serum amylase, lipase, and trypsin levels in a subset of responders to therapy (n=4-6), placebo “responders” (n=2), treated non-responders (n=16), and placebo non-responders (n=10) at baseline, two weeks, three months, and six months after treatment. A two-year sample was not available for this analysis.

### 2.2 Laboratory analysis

Amylase and lipase were quantified under blinded conditions at UF Health Pathology Laboratories (Gainesville, FL) using standard clinical assays involving direct enzymatic colorimetric analysis (Beckman Coulter, Brea, CA) and enzyme-coupled colorimetric methods, respectively. The assay-specific normal serum reference ranges were 29-103 units/L for amylase and 11-82 units/L for lipase. Serum trypsin was measured under blinded conditions at ARUP Laboratories (Salt Lake City, UT) using a standard clinical radioimmunoassay (reference range 115-350 ng/mL). We utilized existing data on β-cell function and glycemia (i.e., area under the curve (AUC) C-peptide during a 2-hour mixed-meal tolerance test at baseline, three months, six months, and two years and HbA1c % at baseline) and de-identified demographic information including age at diagnosis and sex.

### 2.3 Statistical analysis

Data were analyzed and graphed using R Statistical Software. Data are presented as median ± interquartile range (IQR) unless otherwise noted. Kruskal-Wallis and Fisher’s Exact tests were used to compare baseline demographic information and glycemic data. The percent difference from baseline was calculated for amylase, lipase, and trypsin for each individual at two weeks, three months, and six months. Unpaired t-tests compared the percent difference from baseline to 6 months between responders and non-responders to immunotherapy and between treated and placebo participants. A Mann-Whitney test compared exocrine enzyme values at baseline in responders versus non-responders to treatment. A Fisher’s exact test compared the percentage of treated responders versus non-responders having exocrine enzyme values below the reference range at baseline.

## 3 Results

### 3.1 Demographics and glycemic indices

Our subpopulation had a median age of 15 years and was 44% female, which was consistent across groups (**Table 1**). 2-hour AUC C-peptide was higher in responders versus non-responder and placebo participants at baseline (p=0.03), six months (p=0.003), and two years (p<0.001).

**Table 1:** Participant demographics and characteristics.

	Responders (n=4-6; 67% ATG-treated)	Non-responders (n=16; 62.5% ATG-treated)	Placebo “Responders” (n=2)	Placebo Non-responders (n=10)	Overall (n=34)	P-value
Age (years)	13.5 (13,17)	15.5 (14,16)	19 (15,23)	16 (15,18)	15 (13,17.3)	0.33
Sex (F,%)	50%	50%	50%	30%	44%	0.65
Baseline HbA1c (%)	6.4 (6,7.1)	7.6 (7.3,8.6)	8.1 (7.6,8.6)	7.5 (6.7,9)	7.5 (6.9,8.6)	0.1
Baseline AUC C-peptide (nmol/L)	0.65 (0.31,0.67)	0.28 (0.2,0.39)	0.24 (0.18,0.29)	0.41 (0.34,0.48)	0.33 (0.24,0.48)	0.03
6 mo. AUC C-peptide (nmol/L)	0.61 (0.41,0.72)	0.18 (0.13,0.27)	0.25 (0.23,0.28)	0.25 (0.19,0.3)	0.25 (0.15,0.34)	0.003
2 yr. AUC C-peptide (nmol/L)	0.48 (0.39,0.58)	0.1 (0.06,0.11)	0.19 (0.12,0.26)	0.13 (0.07,0.17)	0.12 (0.07,0.28)	<0.001

Values reported in median (interquartile range) unless otherwise noted.

Responders to immunotherapy were defined as participants with at least 60% of baseline area under the curve (AUC) C-peptide levels after a 2-hour mixed meal tolerance test (MMTT) at 2 years post-treatment. Participants were treated with either low-dose anti-thymocyte globulin (ATG), low-dose ATG + pegylated granulocyte colony-stimulating factor (GCSF), or placebo.

### 3.2 Amylase

**Figure 1:**
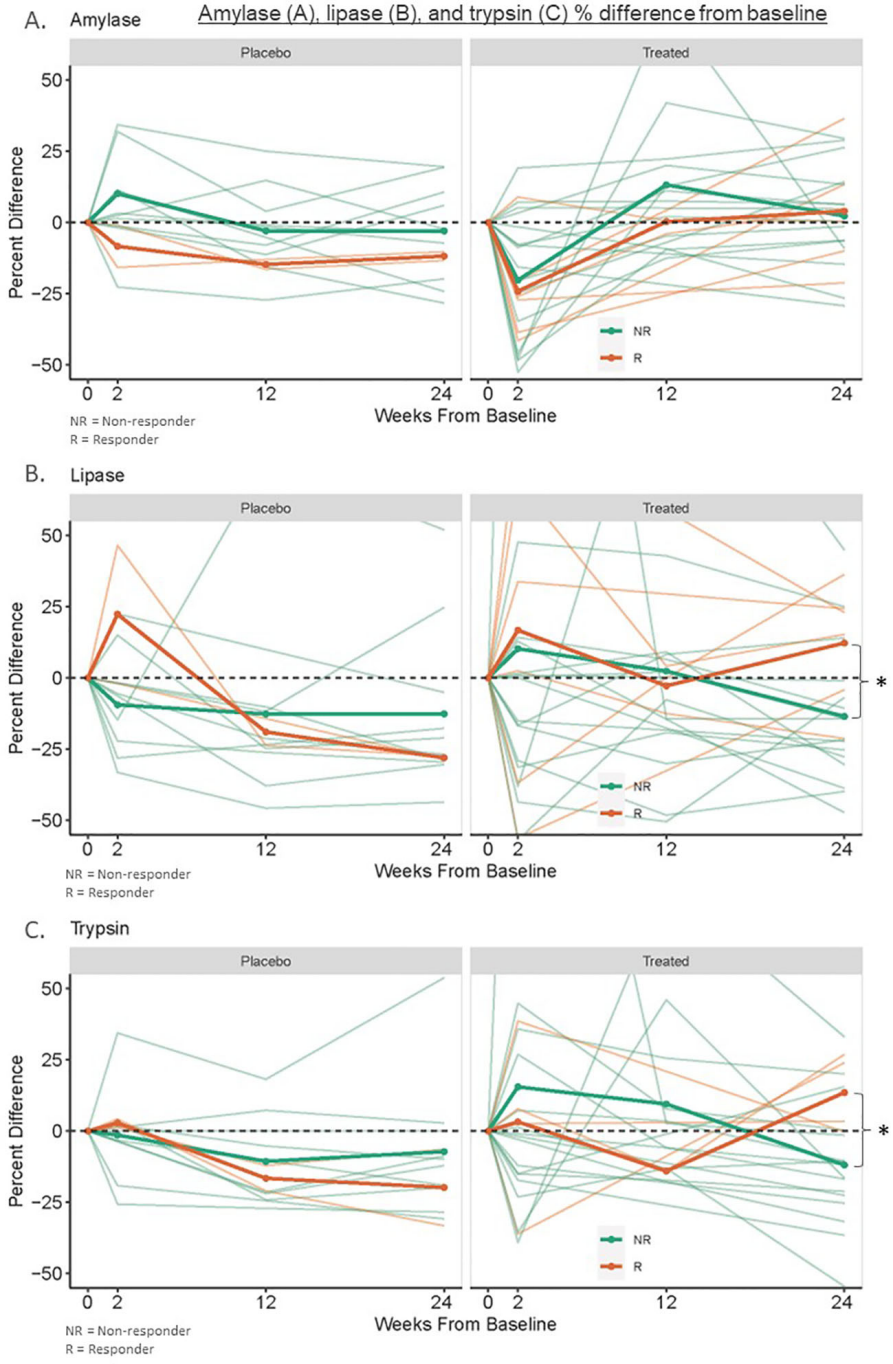
Amylase **(A)**, lipase **(B)**, and trypsin **(C)** % difference from baseline. This was measured in banked serum of new-onset type 1 diabetes treated responders (n=4-6), treated non-responders (n=16), placebo “responders” (n=2), and placebo non-responders (n=10) to therapy with ATG or ATG/GCSF. Responders to immunotherapy were defined as participants with at least 60% of baseline area under the curve (AUC) C-peptide levels after a 2-hour mixed meal tolerance test (MMTT) at 2 years post-treatment. *Treated responders had 6-month lipase and trypsin levels 116% and 115% of baseline while non-responders had levels 80% and 91% of baseline respectively (p=0.03).

No participant had an elevated amylase above the reference range at any measured point (baseline, 2 weeks, three months, or six months). Thirty-five percent of amylase values were below the reference range at baseline (**Table 2**). There were no significant trends in longitudinal amylase values post-treatment (**Figure 1**). There was no statistical difference between baseline amylase values (p=0.91) or the percentage of participants with an amylase value below the reference range at baseline among responders versus non-responders to immunotherapy (p=1.0). Responder (n=6) six-month amylase levels were 104% of baseline versus placebo “responders” (n=2) at 102%, and placebo non-responders (n=10) and treated non-responders (n=16) at 88% and 97% of baseline (p=0.86 for responders versus non-responders to immunotherapy; p=0.26 for treated versus placebo participants).

**Table 2:** Amylase values at baseline and 6 months.

	Responders (n=6; 67% ATG-treated)		Non-responders (n=16; 62.5% ATG-treated)		Placebo (n=12)		Overall (n=34)	
	Amylase (U/L)	% Below RR	Amylase (U/L)	% Below RR	Amylase (U/L)	% Below RR	Amylase (U/L)	% Below RR
Baseline	32.8 (28, 34.9)	33%	32.7 (24.1, 42.5)	38%	30.9 (27.8, 41.8)	33%	31.6 (26.7, 38.6)	35%
6 months	31.2 (26.2, 40.7)	50%	33.5 (23.9, 41.8)	44%	31.6 (24.7, 40.8)	33%	32.7 (25.5, 41.3)	41%

Values reported in median (interquartile range) unless otherwise noted. RR, reference range.

Responders to immunotherapy were defined as participants with at least 60% of baseline area under the curve (AUC) C-peptide levels after a 2-hour mixed meal tolerance test (MMTT) at 96 weeks post-treatment. Participants were treated with either low-dose anti-thymocyte globulin (ATG), low-dose ATG + pegylated granulocyte colony-stimulating factor (GCSF), or placebo. The reference range for amylase was 29-103 U/L.

### 3.3 Lipase

One ATG-only treated non-responder participant had a lipase level slightly above the reference range at three months (88 U/L, RR 11-82 U/L); no other participants had an elevated lipase level at any measured point. Thirty-five percent of lipase values were below the reference range at baseline (**Table 3**). Baseline lipase values were lower but not statistically different in responders versus non-responders (p=0.08). Similarly, a greater percentage of responders had lipase levels below the reference range compared to non-responders, but this was not statistically significant (p=0.18). Responder (n=6) 6-month lipase levels were higher at 116% of baseline compared to placebo “responders” (n=2), placebo non-responders (n=10), and treated non-responders (n=16) which were at 72%, 90%, and 80% of baseline, respectively (p=0.03 for responders versus non-responders to immunotherapy; p=0.36 for treated versus placebo participants) (**Figure 1**).

**Table 3:** Lipase values at baseline and 6 months.

	Responders (n=6; 67% ATG-treated)		Non-responders (n=16; 62.5% ATG-treated)		Placebo (n=12)		Overall (n=34)	
	Lipase (U/L)	% Below RR	Lipase (U/L)	% Below RR	Lipase (U/L)	% Below RR	Lipase (U/L)	% Below RR
Baseline	9.2 (8, 14.4)	67%	15.3 (9.9, 19.8)	31%	13.6 (11.6, 16.9)	25%	14.2 (9.9, 17.7)	35%
6 months	10 (9.2, 16.6)	33%	11.4 (9.1, 16.6)	44%	11.6 (9.4, 14.3)	42%	11.2 (9.2, 15.8)	47%

Values reported in median (interquartile range) unless otherwise noted. RR, reference range.

Responders to immunotherapy were defined as participants with at least 60% of baseline area under the curve (AUC) C-peptide levels after a 2-hour mixed meal tolerance test (MMTT) at 96 weeks post-treatment. Participants were treated with either low-dose anti-thymocyte globulin (ATG), low-dose ATG + pegylated granulocyte colony-stimulating factor (GCSF), or placebo. The reference range for lipase was 11-82 U/L.

### 3.4 Trypsin

Two responders did not have sufficient serum quantity to run trypsin levels, so four responder participants were analyzed. Two ATG-only treated non-responder participants and one placebo non-responder had trypsin levels above the reference range at various times (median of these participants 389 ng/mL, IQR 222-488, RR 115-350 ng/mL). Only one trypsin level at three months in an ATG-treated non-responder reached a cutoff potentially concerning for acute pancreatitis (751 ng/mL) (6), however, their concurrently measured amylase and lipase values were reassuring (68 U/L (RR 29-103) and 88 U/L (RR 11-82) respectively). Sixteen percent of trypsin values were below the reference range at baseline (**Table 4**). Baseline trypsin levels in responders to immunotherapy were lower than in non-responders, but this was not statistically significant (p=0.3). There was no difference in the percentage of participants with a trypsin level below the reference range at baseline among responders versus non-responders to immunotherapy (p=0.5). Responders to immunotherapy (n=4) had 6-month trypsin levels at 115% of baseline vs placebo “responders” (n=2), placebo (n=10), and non-responders (n=16) at 85%, 91%, and 91% of baseline respectively (p=0.03 for responders versus non-responders to immunotherapy; p=0.78 for treated versus placebo participants) (**Figure 1**).

**Table 4:** Trypsin values at baseline and 6 months.

	Responders (n=4; 50% ATG-treated)		Non-responders (n=16; 62.5% ATG-treated)		Placebo (n=12)		Overall (n=32)	
	Trypsin (ng/mL)	% Below RR	Trypsin (ng/mL)	% Below RR	Trypsin (ng/mL)	% Below RR	Trypsin (ng/mL)	% Below RR
Baseline	132.6 (123.1, 161.9)	25%	153.8 (136, 187)	13%	144.3 (128.1, 212.5)	17%	145.4 (129, 196)	16%
6 months	139.5 (134.7, 191.7)	0%	123.6 (110.3, 176)	44%	132.5 (103.5, 218.1)	42%	133.1 (111.7, 190.2)	34%

Values reported in median (interquartile range) unless otherwise noted. RR, reference range.

Responders to immunotherapy were defined as participants with at least 60% of baseline area under the curve (AUC) C-peptide levels after a 2-hour mixed meal tolerance test (MMTT) at 96 weeks post-treatment. Participants were treated with either low-dose anti-thymocyte globulin (ATG), low-dose ATG + pegylated granulocyte colony-stimulating factor (GCSF), or placebo. The reference range for trypsin was 115-350 ng/mL.

## 4 Discussion

Our study suggests that two serum exocrine enzymes, lipase and trypsin, may be useful as biomarkers of immunotherapy responses in patients with new-onset T1D. In our study, six months after treatment, lipase and trypsin increased to 115% of baseline in new-onset T1D responders to immunotherapy but declined to 80-90% of baseline in non-responders and placebo participants. This difference was not present before the six-month time point. This is consistent with what has been reported regarding C-peptide differences after T1D immunotherapy, where a significant treatment effect predicting long-term C-peptide preservation is not seen until six months post-therapy (21). Additionally, in our study, no differences were seen in longitudinal amylase values. This may be due to the higher specificity of lipase and trypsin for pancreatic pathology and fits with previous studies showing decreases in lipase and trypsin along the continuum of early T1D progression with amylase only reduced in clinical disease (2). Baseline levels of lipase and trypsin tended to be lower in responders to treatment versus non-responders; however, these variations were not significant in this small sample. Thirty-five percent of new-onset T1D participants had low amylase and lipase levels, and 16% had low trypsin levels at baseline, which is in agreement with previous studies (2, 11). Only one participant had an elevated trypsin level more than twice the upper limit of normal three months after receiving ATG, with concurrently measured amylase and lipase levels not consistent with acute pancreatitis (22). As the two week post-treatment value in this participant was within the normal reference range, this was very unlikely to be related to treatment (23). Our findings were limited by the small sample size and analysis specific to treatment with ATG and ATG/GCSF; further studies with larger numbers of participants are warranted to determine their predictive utility.

Six-month differences in lipase and trypsin from baseline may be beneficial for early identification of response to T1D immunotherapy. These serum markers should be further evaluated both individually and in combination with immune signatures and other differentiating serologic features. If our findings are replicated in larger populations, the measurement of serum exocrine enzymes could be part of a broader T1D immunotherapy individualized treatment strategy (24). In this era of emerging immunotherapeutic options to treat T1D, the early identification of response to therapy could allow for individualized decision-making regarding retreatment or treatment with another therapy and could inform adaptive trial designs (17). Additionally, possibly lower baseline values of lipase and trypsin in responders to ATG or ATG/GCSF should be further explored. A baseline signature predicting response to immunotherapy could be beneficial for improving clinical trial recruitment design and developing future individualized treatment plans (16).

This study did not evaluate other exocrine biomarkers previously identified as abnormal in pre-clinical T1D, including fecal elastase, serum carboxypeptidase A1 and chymotrypsinogen B1, and pancreas volume by MRI (7–11, 14, 15). These markers, along with a more comprehensive analysis of exocrine pancreatic protein expression, could also be helpful in evaluating response pre- and post-T1D immunotherapy. However, circulating serum lipase and trypsin have the advantage of being clinically available and easily collected along with other risk indices with low cost and high compliance.

While reduced exocrine pancreatic function at T1D disease onset has been well established, the mechanism remains largely unknown (13). The normal increase in lipase and trypsin in responders to immunotherapy could be due to a direct improvement in the autoimmune process influencing exocrine function or an indirect effect from improved β-cell function. Further experiments in preclinical models, and imaging to measure pancreas volume in future clinical studies, would be needed to determine whether this effect is mediated by an increase in pancreatic size, transcription of exocrine enzymes or prevention of their degradation, or increased release into the circulation. However, it was interesting that baseline levels of lipase and trypsin were not higher and were possibly lower in responders to immunotherapy. We had expected that the relatively higher baseline β-cell function in these individuals would be correlated with improved baseline exocrine serologic markers since many hypothesize that insulinopenia is a significant contributor to exocrine insufficiency in T1D (18). This finding should be further evaluated in a larger cohort. Our findings provide preliminary evidence that exocrine enzymes may be valuable biomarkers of response to immunotherapy in T1D and should drive future lines of inquiry in this novel and yet unexplored area.

## Data Availability

The raw data supporting the conclusions of this article will be made available by the authors, without undue reservation.
